# Reliability and reproducibility of individual differences in functional connectivity acquired during task and resting state

**DOI:** 10.1002/brb3.456

**Published:** 2016-03-30

**Authors:** Lubdha M. Shah, Justin A. Cramer, Michael A. Ferguson, Rasmus M. Birn, Jeffrey S. Anderson

**Affiliations:** ^1^ Department of RadiologyUniversity of UtahSalt Lake CityUtah84132; ^2^ Department of BioengineeringUniversity of UtahSalt Lake CityUtah84132; ^3^ Department of PsychiatryUniversity of WisconsinMadisonWisconsin 53705

**Keywords:** Functional connectivity, resting state fMRI reproducibility

## Abstract

**Objectives:**

Application of fMRI connectivity metrics as diagnostic biomarkers at the individual level will require reliability, sensitivity and specificity to longitudinal changes in development, aging, neurocognitive, and behavioral performance and pathologies. Such metrics have not been well characterized for recent advances in BOLD acquisition.

**Experimental Design:**

Analysis of multiband BOLD data from the HCP 500 Subjects Release was performed with FIX ICA and with WM, CSF and motion parameter regression. Analysis with ROIs covering the gray matter at 5 mm resolution was performed to assess functional connectivity. ROIs in key areas were used to demonstrate statistical differences between specific connections. Reproducibility of group‐mean functional connectivity and for single connections for individuals was evaluated for both resting state and task acquisitions.

**Principal Observations:**

Systematic differences in group‐mean connectivity were demonstrated during task and rest and during different tasks, although individual differences in connectivity were maintained. Reproducibility of a single connection for a subject and across subjects for resting and task acquisition was demonstrated to be a linear function of the square root of imaging time. Randomly removing up to 50% of time points had little effect on reliability, while truncating an acquisition was associated with decreased reliability. Reliability was highest within the cortex, and lowest for deep gray nuclei, gray‐white junction, and near large sulci.

**Conclusions:**

This study found systematic differences in group‐mean connectivity acquired during task and rest acquitisions and preserved individual differences in connectivity due to intrinsic differences in an individual's brain activity and structural brain architecture. We also show that longer scan times are needed to acquire data on single subjects for information on connections between specific ROIs. Longer scans may be facilitated by acquisition during task paradigms, which will systematically affect functional connectivity but may preserve individual differences in connectivity on top of task modulations.

## Introduction

Functional connectivity MRI is an evolving, diversely utilized technology for identifying brain network organization (Fox and Raichle 2007; Biswal et al. 2010; Van Dijk et al. 2010). There are an increasing number of applications, including characterization of neurodevelopmental and neuropsychological conditions based on differences in brain network architecture and functional network connectivity. Most studies to date have used group means in specific or aggregate metrics of functional connectivity between different regions of the brain to identify how a disorder may have altered brain connectivity. Nevertheless, group mean differences are difficult to apply in a clinical setting in which one is concerned with diagnosis, prognosis, and treatment monitoring in a single patient. For clinical applications, it is important for functional connectivity researchers to develop techniques that have single‐subject specificity and those that may be performed in a variety of acquisition strategies, such as task‐based connectivity or with variable scan lengths. As longitudinal biomarkers, functional connectivity metrics need to be reliable as well as sensitive and specific to longitudinal changes (Dosenbach et al. 2010; Satterthwaite et al. 2012).

Currently, no functional connectivity MRI test has found widespread clinical use for diagnosis or prognosis of an individual patient, despite the many disorders for which group differences in connectivity have been hypothesized to play a role in pathophysiology (Castellanos et al. 2013). Underlying this fact are fundamental unresolved questions: Is the information contained in functional connectivity metrics simply too variable to serve as a sensitive and specific clinical assay for neurological and neuropsychiatric disorders in individual patients? Are normal differences in connectivity between individuals greater than differences associated with disorders or prognostic subgroups? If not, are there solutions to technical factors that would allow functional connectivity MRI to become a clinically relevant test?

Answering these questions requires detailed information about the reliability and reproducibility of functional connectivity MRI. In order to distinguish individuals with a neurological, psychiatric, or developmental condition, the reliability of measurement must be more robust than expected alterations in connectivity attributable to the condition and normal variation within a healthy population. Previous studies have suggested a relatively robust test–retest reliability even for relatively short duration functional MRI scans (Shehzad et al. 2009; Van Dijk et al. 2010), with improved reliability discriminating individual connections of a single subject from a group of healthy control subjects for longer scans (Anderson et al. 2011; Birn et al. 2013). A recent multisite analysis on the test–retest reliability of voxel‐wise metrics of seven common brain networks revealed that the default, control, and attention networks were most reliable (Zuo and Xing 2014). The study also found that ICA (independent component analysis) with dual regression, local functional homogeneity, and functional homotopic connectivity were the three mostly reliable resting state fMRI metrics. But many of these metrics tested are relatively coarse, such as which brain regions lie in which networks. Clinical applications are likely to be much more regionally specific, evaluating differences in precise brain circuits.

Moreover, all the previous work characterizing reproducibility of functional connectivity has used older methodological paradigms with conventional BOLD pulse sequences for analysis. Recent functional MRI data acquisition advancements include multiband echoplanar sequences, longer imaging times, and lower repetition times (TR), which may allow dramatic improvements in single subject characterization. The HCP (Human Connectome Project) datasets (RRID:SCR_003922; http://www.humanconnectome.org/documentation/S500/) is the largest publicly available functional connectivity datasets with such higher spatial and temporal resolutions (Glasser et al. 2013). The differences in technique are not subtle changes. Modern acquisitions may have an order of magnitude more data per subject than has been typically acquired, with higher image quality and methodological homogeneity. The HCP dataset affords an opportunity to ask not only whether recent advancements have crossed the threshold where individual differences are approachable but also what specific technical factors may show promise for further optimization.

We analyzed the HCP dataset with two aims. First, can individual‐specific functional connectivity differences be identified with HCP‐type protocols, and what imaging times may be required to achieve this goal. With the advent of multiband acquisitions with higher temporal sampling rates, it is also unclear whether such scans show improvements in reliability and reproducibility given higher number of volumes acquired per minute, or whether increased temporal resolution may have diminishing returns given the slow frequencies underlying functional connectivity effects (Cordes et al. 2001). Methodological concerns such as whether motion censoring or scrubbing (Power et al. 2012) should be performed are a related issue, since removing time points from an acquisition may decrease scan length and introduce discontinuities that decrease reliability and reproducibility.

The second aim of this study was to ascertain whether individual differences in functional connectivity between brain regions are preserved during task and resting acquisitions. It has been proposed that a “resting state” may be an elusive concept, resulting in a wider variance of cognitive state and therefore brain connectivity, than during a focused task (Morcom and Fletcher 2007; Perrin et al. 2012). This may be advantageous, or not, given a desire to measure many diverse brain states versus sampling more homogenous brain states with decreased variance of functional connectivity (Buckner and Vincent 2007). If imaging acquisition time is a critical factor in allowing functional connectivity MRI to approach clinical utility as hypothesized in aim 1, strategies to allow longer acquisitions may be important. Longer scan times pose risks of the subject falling asleep (Tagliazucchi and Laufs 2014) and nonstationarity from cognitive fluctuations (Buckner et al. 2013). Adding task acquisitions can lengthen scan periods with more homogenous cognitive states that hold a subject's attention (Vanderwal et al. 2015). Functional connectivity can be also be measured while acquired in a nonresting state and may facilitate detection of weaker connections (Bartels and Zeki 2005; Hasson et al. 2009; Vanderwal et al. 2015).However, the question becomes whether task acquisition results in similar functional connectivity data as that obtained during resting state. While it is clear that audiovisual stimuli and task conditions may drive systematic differences in functional connectivity that may be largely shared across individuals, it is not known whether individual differences in connectivity may be expected to be similar to those acquired during “resting state.”

## Materials and Methods

Analysis of multiband BOLD data from the HCP 500 Subjects Release was performed with two parcellations of the brain. A higher resolution parcellation (6923 regions covering the gray matter at 5 mm resolution) allowed for analysis of functional connectivity in precise circuits, and a coarser parcellation consisting of 264 regions in strategic locations within canonical brain networks allowing assessment of clinically relevant connections (Dosenbach et al. 2010; Power et al. 2012). Reproducibility of group‐mean functional connectivity and for single connections for individuals was evaluated for resting state and task acquisitions. Additionally, the effect of systematically varying the amount of data was assessed using separate strategies with simulated scrubbing.

### Dataset studied

We analyzed data from 476 subjects from the Human Connectome Project 500 Subjects Release. The MRI sequences in the HCP include diffusion imaging, resting‐state fMRI, task‐evoked fMRI, and T1‐ and T2‐weighted MRI for structural and myelin mapping (Detail of the parameters are include in the Appendix S1). The multiband BOLD resting state data released includes both minimally processed as well as FIX ICA cleaned (Feinberg et al. 2010; Moeller et al. 2010; Setsompop et al. 2012; Xu et al. 2012; Glasser et al. 2013; Van Essen et al. 2013; Griffanti et al. 2014) to allow for evaluation of the impact of preprocessing pipelines on results (Glasser et al. 2013).

Subjects ranged from 22 to 35 years in age, with 280 female and 196 male participants. These subjects comprised all those for whom four 15‐min resting BOLD sequences were available. Minimally processed task‐based fMRI data were also used for analysis for each of the seven task sequences for each subject for whom two acquisitions of the task were available (emotion task: *n* = 471; gambling task: *n* = 469; language task: *n* = 470; motor task: *n* = 474; relational task: *n* = 468; social task: *n* = 474; working memory task: *n* = 474).

### Preprocessing

Minimally preprocessed data (Glasser et al. 2013) were used to allow a similar preprocessing strategy for BOLD data acquired during resting state and during seven task acquisitions with the following preprocessing steps:

A gray matter mask was compiled from skull stripped BOLD images for all 476 subjects showing voxels where an a priori gray matter mask (grey.nii, SPM 12b (RRID:SCR_007037; http://www.fil.ion.ucl.ac.uk/spm/) (Evans et al. 1992) were inside the brain for 95% of subjects. This image was parcellated into 6923 nonoverlapping 5‐mm diameter ROIs covering the cortical and subcortical gray matter. The 6923 ROIs were chosen to represent a parcellation of the gray matter with spatial resolution of 5 mm (Ferguson and Anderson 2012). Specifically, each voxel was tested in sequence beginning with the inferior left voxel in the cerebellum. If a voxel was less than 5 mm distant to voxels already selected, then this voxel was included in the set of ROI center coordinates. When all voxels had been tested, 6923 voxels remained, and gray matter voxels were parcellated based on which of the 6923 center coordinates was closest to a given voxel.

1. The MPRAGE image for each subject was segmented using SPM12b into gray matter, white matter, and CSF images, and a mask was created for each subject by thresholding these images at 0.5. This mask was degraded for CSF and WM by eliminating all voxels that were not surrounded on all sides by CSF and WM voxels in the mask.
2. A bandpass filter was applied (idealfilter.m, MATLAB (RRID:SCR_001622; http://www.mathworks.com/products/matlab/) between 0.001 and 0.1 Hz, and each time series was subjected to a linear detrend operation in conjunction with the WM, CSF, and motion regression (each time series and covariate was detrended and bandpass filtered prior to regression).
3. Time series for WM, CSF, and 12 detrended motion parameters supplied with the HCP dataset were regressed from the BOLD time series for each of the 6923 gray matter ROIs to mitigate the effects of physiological noise in the fMRI data. The first 20 volumes of each resting state sequence were discarded, leaving 1180 volumes per sequence of resting state data. For task data, the first 20 volumes were also discarded for each sequence. Motion scrubbing (Power et al. 2012) was not used for any of the results to allow for systematic variation in the length of time series across subjects and its effect on reliability.
4. Fisher‐transformed correlation coefficients representing functional connectivity were extracted for each pair of 6923 × 6923 ROIs separately for each of the four resting state sequences. This resulted in 23,960,503 connections for each sequence analyzed.

Additionally, FIX ICA‐cleaned BOLD resting state data (Griffanti et al. 2014) were used without additional preprocessing steps, to calculate Fisher‐transformed correlation coefficients for the same 6923 × 6923 ROIs for each resting state acquisition.

### Seed‐based analyses

Analysis of the fMRI data was performed using two parcellations. First, a large number of regions (6923 ROIs covering the cortical and subcortical gray matter) was used to analyze the functional connectivity networks at higher spatial resolution. Second, analysis of a second paracellation of 264 ROIs, a subset of the first, in strategic locations allowed for analyses of statistical differences involving computationally more intensive comparisons.

The Fisher‐transformed correlation coefficients were obtained for each pair of 6923 × 6923 ROIs. These extensive correlation coefficients represent the functional connectivity and were analyzed for the spatial resolution of the functional networks. In order to compare canonical network patterns, five of the 6923 ROIs were selected a priori to represent distinct functional networks: Seed 1, right anterior insula (salience network, MNI coordinates: *x* = 42, *y* = 12, *z* = 10); Seed 2, left posterior cingulate (default mode network, MNI coordinates: *x* = −4, *y* = −50, *z* = 30); Seed 3, right occipital pole (visual network, MNI coordinates: *x* = 14, *y* = −98, *z* = −10); Seed 4, right precentral gyrus (sensorimotor network, MNI coordinates: *x* = 38, *y* = −22, *z* = 60); Seed 5, left frontal eye field (dorsal attention network, MNI coordinates: *x* = 25, *y* = −4, *z* = 50). Connectivity of the other 6922 ROIs to each of the five seed ROIs was used to obtain snapshots of core canonical resting state networks with different preprocessing strategies, in resting state acquisition data, and in task acquisition data for each sequence analyzed.

To compare a subset of ROIs strategically positioned in nodes of major networks, a subset of 264 ROIs was used from previous literature reports (Dosenbach et al. 2010; Power et al. 2011, 2012) by identifying which of the 6923 ROIs corresponded to MNI coordinates of the 264 ROIs reported by Power et al. This subanalysis for statistical differences in group‐mean connectivity mitigates the effects of multiple comparisons. The 264 ROIs also allow for computational tractability of correlation coefficients rather than the 23.9 million connections obtained using the 6923 ROIs and allow reliability and reproducibility calculations to a meaningful subset of strategically positioned ROIs that are likely to reflect functional connections of interest.

To evaluate for differences in reliability associated with segmentation of ROIs, we additional extracted resting state time series from fourteen subject‐specific subcortical regions using Freesurfer‐derived segmentation (Fischl et al. 2002) of bilateral thalami, caudate nuclei, putamen, amygdalae, hippocampi, pallidum, and nucleus accumbens. The time series was averaged for all voxels within each of these 14 ROIs for each subject.

### Reliability and reproducibility analyses

Intraclass correlation coefficients (ICC.m in MATLAB) were used to compare reliability of functional connectivity of each of the 6923 ROIs to each of the 264 ROI subset for each subject in the four 15‐min acquisitions. For each of the 6923 ROIs, this allowed calculation of a mean ICC for the connections across the strategic 264 ROI subset and across subjects. Display of the mean ICC for each of the 6923 ROIs allows display of a 5 mm resolution map of functional connectivity reliability. We also calculated ICC for connections between each of the 14 subcortical ROIs and the same 264 ROI subset to evaluate for differences in ICC associated with segmentation of larger ROIs defined by subject‐specific structural gray matter boundaries rather than MNI coordinates.

Intraclass correlation coefficients for each ROI in each subject was also calculated after systematically varying the amount of data present using two separate strategies. First, a “simulated scrubbing” was performed where randomly selected datapoints were removed from the time series at each ROI and the remaining time points concatenated, analagous to a scrubbing procedure to delete high motion timepoints (Power et al. 2012). Second, the scans were truncated by only including timepoints from the beginning of the scan. Using both methods, ICC calculations were repeated for time series that were missing 0%, 5%, 10%,… through 50% of the data.

Reproducibility of group mean connectivity between task and rest acquisitions values for 6923 × 6923 ROIs were assessed using correlation coefficient across the 23.9 million connections in resting state (average of all four acquisitions for 476 subjects) and for each task (average of two acquisitions for 476 subjects).

For each individual connection, the root‐mean‐square difference between two acquisitions of the task or resting state data in Fisher‐transformed correlation was used as an estimate of the reproducibility. When averaged across all connections this yielded an estimate of the likely uncertainty in Fisher‐transformed correlation for an individual functional connectivity measurement between two 5‐mm diameter ROIs.

To evaluate the reproducibility of subject‐specific differences in functional connectivity, correlation coefficient across subjects was calculated for each individual connection acquired during two different task or resting acquisitions. This correlation coefficient was averaged across connections after Fisher transformation. If the same subjects showed relatively higher connectivity for a particular connection in both task and rest scans, this would be evidence that individual differences in connectivity persist during rest and task acquisitions. Furthermore, this would also show that, in the same task conditions, differences in connectivity for that individual compared to other subjects may be expected to be similar regardless of the task condition for which the data were acquired.

## Results

### Effects of preprocessing methods on fMRI data analysis

The WM, CSF, motion‐regressed method resulted in slightly higher Fisher‐transformed correlations of group mean connectivity for each pair of 6923 × 6923 ROIs for each of the four resting state sequences as compared to the correlation coefficients for the same ROIs for each resting state acquisition FIX ICA‐cleaned BOLD resting state data. However, the overall spatial pattern of seed‐based functional connectivity between the diffferent preprocessing strategies was similar (Fig. S1). The FIX ICA method preprocessing strategy yielded systematically reduced connectivity but preserved spatial distribution across five distinct functional networks: salience, default mode, visual, sensorimotor, and dorsal attention in resting state and in task acquisition (Fig. S2).

### Systematic differences in functional connectivity between task and resting state acquisitions

To look at differences in group mean functional connectivity between resting state and task acquisitions, a parcellation of 6923 regions of interest covering the gray matter was used, with measurements of connectivity between each pair of ROIs. This resulted in 23.9 million connections between ROIs. Differences in resting state compared to task‐driven functional connectivity were measured by subtracting Fisher‐transformed correlation for rest versus task acquisitions for each connection, and a two‐tailed t‐test was performed across 476 subjects to test whether the difference was significantly different from 0. To account for multiple comparisons, a false discovery rate of *q* < 0.05 was applied to determine which connections differed between task and rest. A difference in connectivity of ±0.12 in Fisher‐transformed correlation units corresponded to a significant difference between task and rest acquisitions.

**Figure 1:**
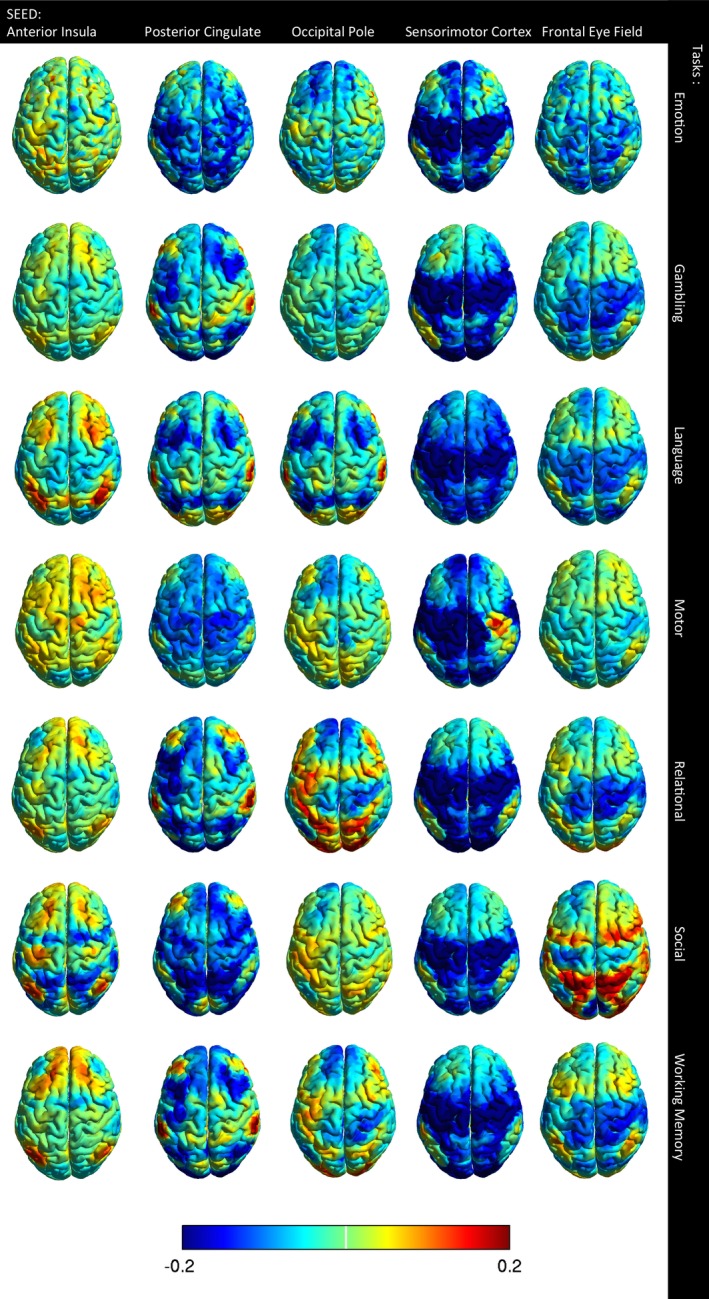
Effect of task on connectivity. The columns indicate the five functional network seeds and the rows indicate seven tasks. Image color shows the difference in connectivity between task and rest for each seed to 6922 other brain regions. A difference in connectivity of ±0.12 is considered significant, corresponding to *q* < 0.05, False Discovery Rate.

Functional connectivity for five seeds corresponding to different functional networks to the rest of the brain had a similar spatial distribution for corresponding seeds in task and rest conditions (Fig. 1). Increased connectivity was observed with most tasks in the salience and visual networks. The default mode network and sensorimotor cortex revealed decreased connectivity with most tasks. The visual network demonstrated increased connectivity during the relational and social tasks with predominantly decreased connectivity for the remaining tasks. Similarly, attention networks showed increased connectivity during the relational, social and working memory tasks. In general, networks expected to be engaged by a task showed higher connectivity during the task acquisition. Gorgolewski et al. also showed inhomogeneous correlations through the whole brain but higher time series correlations for activated regions (Gorgolewski et al. 2013).

But there are exceptions where a task may not result in higher connectivity within a related network. For example, during the motor task, regions of the sensorimotor network were often anticorrelated due to the temporal structure of the task. The motor task involves alternating motor activity in ipsilateral and contralateral body regions, so that when one part of the motor cortex is active, other regions of the motor cortex may not be. With unilateral seed placement in the motor cortex, the signal is the sum of the resting fMRI signal and the forced timing of the task. In addition to connectivity changes reflecting the temporal structure of the task, there are also network‐level changes where a network that participates in a given task may show higher connectivity, for example, the dorsal attention network during a working memory task.

### Reproducibility of group mean functional connectivity during task and resting state acquisitions

**Figure 2:**
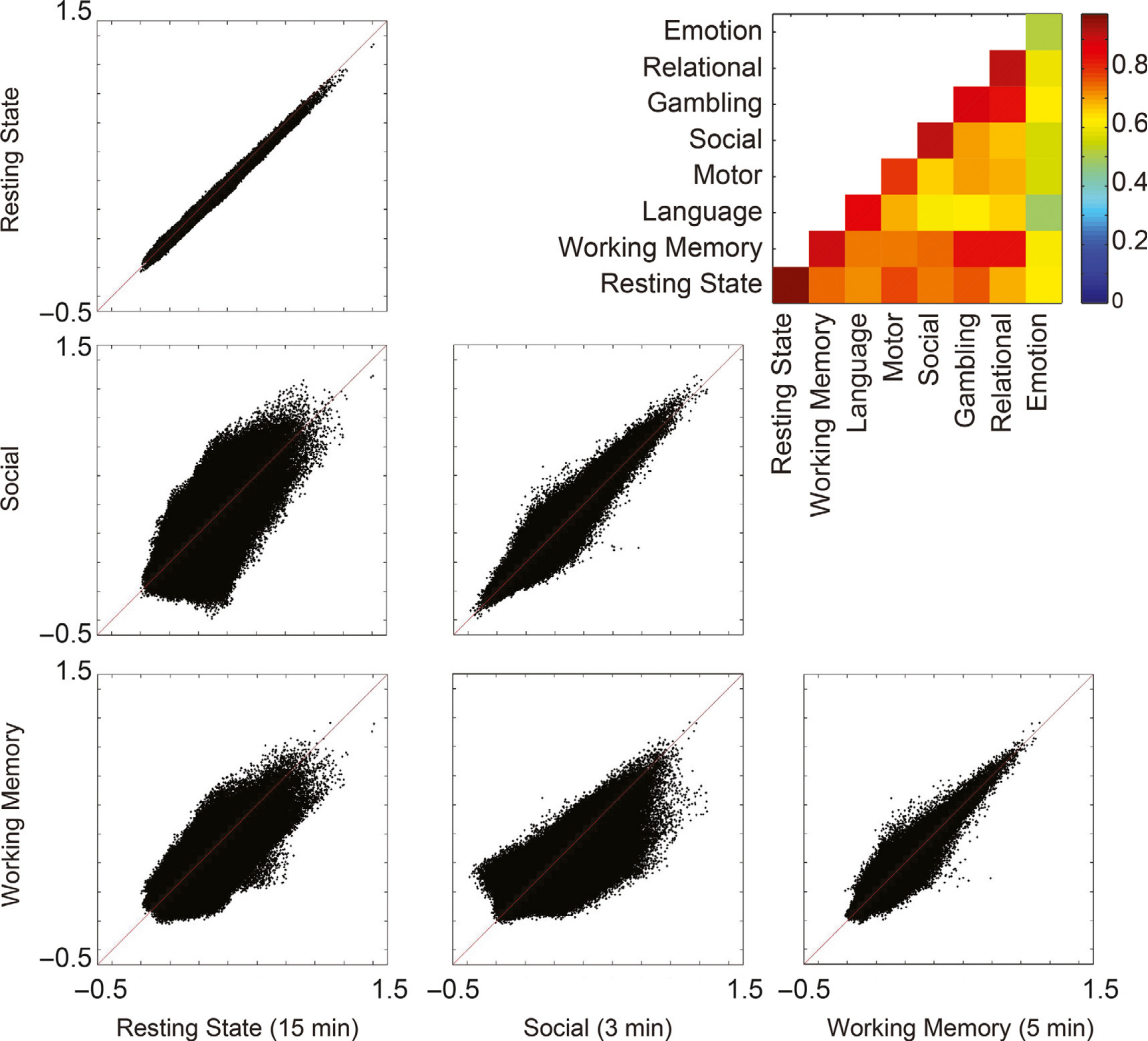
Similarity of group mean connectivity acquired during task and rest. In task and rest, repeat measurements with the same task or rest condition tend toward a constant value, but systematic differences persist between task and rest and between data acquired during different tasks. The inset figure shows correlation coefficient across 6923 × 6923 ROI pairs for group mean results acquired during each condition. The similarity is strongest along the main diagonal, indicating that functional connectivity is more reproducible when acquired with the same task paradigm.

Reproducibility of group mean functional connectivity for both resting state and task acquisitions were measured by evaluating differences between multiple acquisitions of a sequence. Each task acquisition was performed twice in each subject, and each 15‐min resting state acquisition was performed four times for each subject. We compared group mean functional connectivity acquired during one task to functional connectivity acquired during the second acquisition of the same task versus during a different task. Correlation of the group mean connectivity across 23.9 million connections was used as a similarity metric, shown in Figure 2.

During resting state and during task acquisitions, repeat measurements using the same task paradigm tended toward a mean value that lies along the main diagonal when considering a scatter plot of connectivity acquired during two analogous acquisitions. When comparing data acquired during resting state versus task acquisitions, connectivity was much less similar across connections. That is, the repeat measurements using the same task paradigm were more highly correlated than the correlations between different tasks. The systematic differences attributable to different tasks may be related to brain regions artificially synchronized by the timing of task stimuli or cognitive events required by the task. These systematic differences in functional connectivity may be due to the networks engaged in the tasks and the positive or negative correlations of networks as suggested by Figure 1.

The range for correlations of group mean results for repeated runs of the same task was 0.79–0.91 (except for the outlier emotion task at 0.51), with repeated runs of the resting task showing r = 0.95. Comparing group mean results for different tasks ranged from 0.55 to 0.89 (mean 0.71 ± 0.088 SD). The emotion task was the only task for which repeated runs of the same task were less correlated than for comparisons between tasks.

### Spatial distribution of reliablity of functional connectivity during resting state

**Figure 3:**
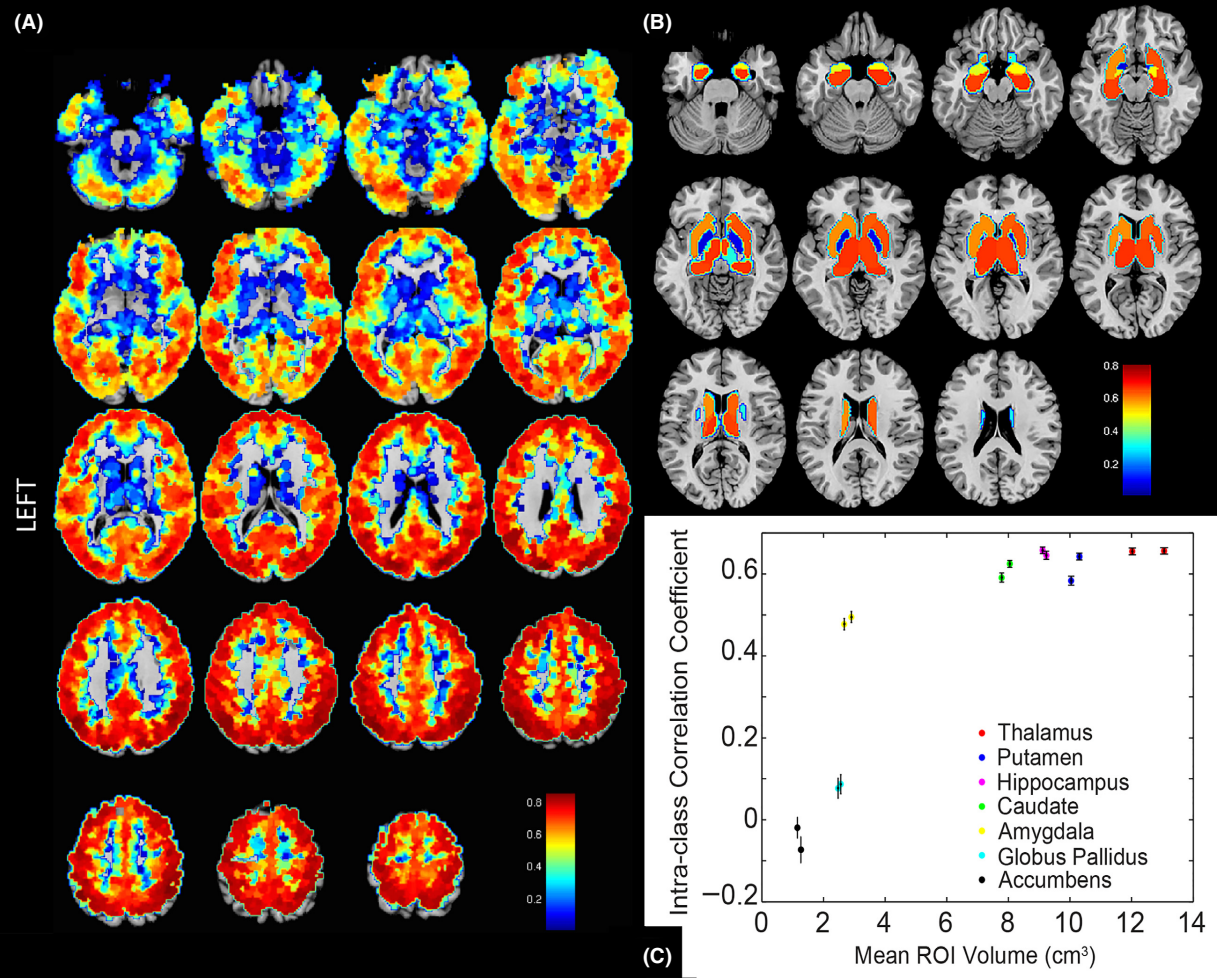
Spatial distribution of the reliablity of functional connectivity. (A) Intraclass correlation coefficients show reliablity for the four 15‐min acquisitons of functional connectivity for each of 6923 ROIs to a subset of 264 strategically positioned ROIs. ICC measurements for 6923 × 264 connections are averaged for each of the 6923 ROIs. Reliablity is highest within the cortex and lower at the gray‐white junction, within the deep gray nuclei, at areas of susceptibility artifact such as the anterior skull base, and at areas near large sulci such as the Sylvian fissure or tentorium cerebelli. Extraction of resting state time series from fourteen subject‐specific subcortical regions using Freesurfer‐derived segmentation (Fischl et al. 2002) of bilateral thalami, caudate nuclei, putamen, amygdalae, hippocampi, pallidum, and nucleus accumbens. The time series was averaged for all voxels within each of these 14 ROIs for each subject. (B) Larger ROIs showed greater reliability, with low reliability measures in bilateral nucleus accumbens, globus pallidi, and amygdalae. (C) Larger ROIs demonstrate higher ICC due to greater volume in the ROI.

For resting state acquisitions, we measured reliabilty of functional connectivity using ICC. This measurement relates the magnitude of measurement error in the functional connectivity measurements in one subject to the inherent variability in functional connectivity measurements between subjects. The mean ICC for each of the 6923 ROIs demonstrates the spatial distribution of functional connectivity reliability, highest within the cortex and lowest for deep gray nuclei, regions at the gray‐white junction, and areas near large sulci such as the Sylvian fissure or tentorium cerebelli, shown in Figure 3A. However, when careful segmentation of subcortical structures was performed, the larger ROIs showed greater reliability (Fig. 3B). There remained low ICC in bilateral nucleus accumbens, globus , and amygdalae. The larger ROIs demonstrate higher ICC due to greater volume (cm^3^) in the ROI (Fig. 3C).

### Effect of scan duration on reproducibility during task and resting state

**Figure 4:**
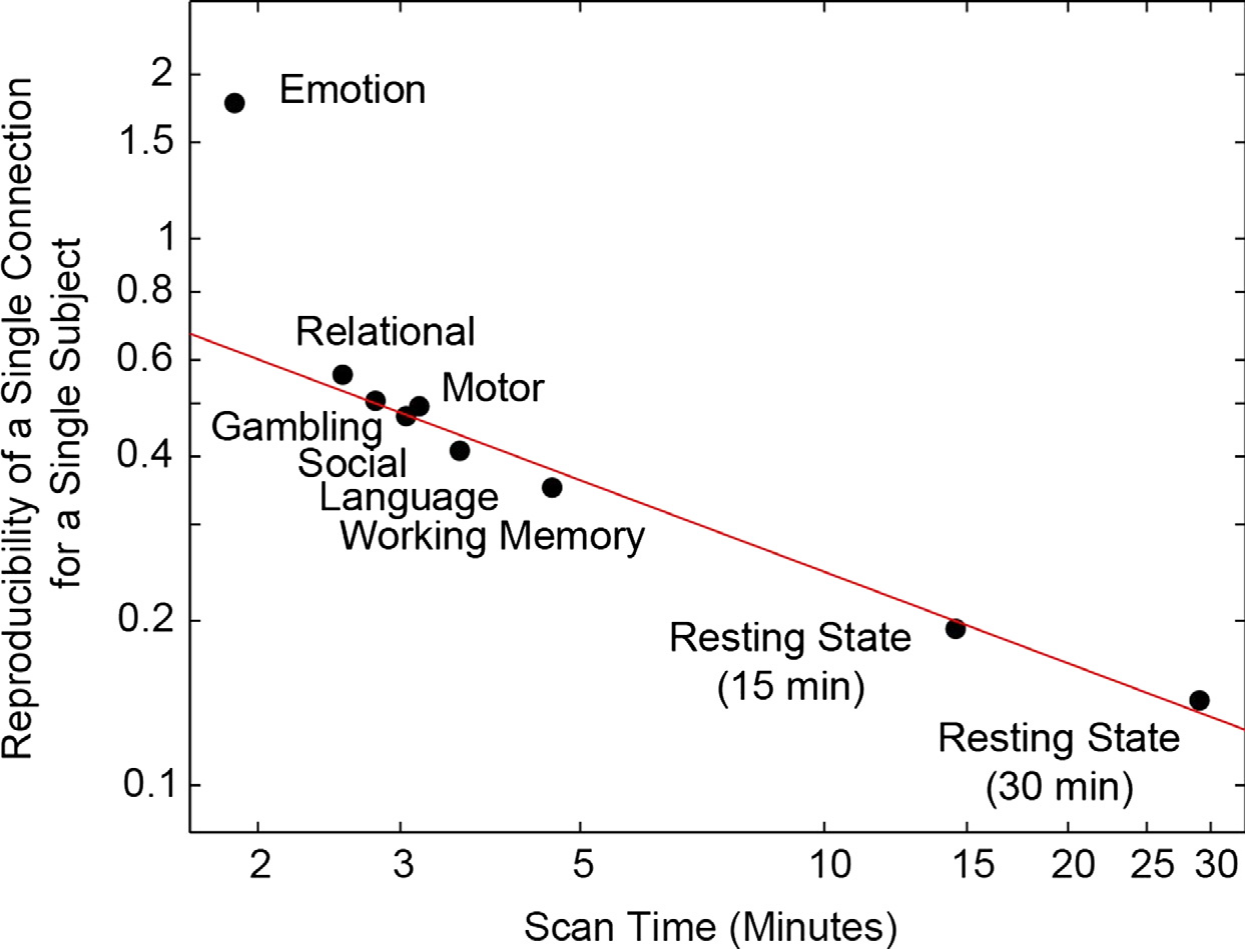
Reproducibility is determined by scan duration. This figure shows the root‐mean‐square difference in connectivity between test and retest scans for connections, averaged over all 6923 × 6923 ROI pairs. Reproducibility is a linear function of the square root of imaging time. The line represents the best fit on a log–log scale and suggests a linear relationship of reproducibility with one over the square root of imaging time. Standard error of the mean is too small to visualize within each data point.

The reproducibility between two acquisitions of the task or resting state data was shown to be a linear function of the square root of imaging time. The reproducibility of a given connection for a single subject was demonstrated by the root‐mean‐square difference in connectivity between test and retest, averaged over all 26 million connections (Fig. 4). The shortest task, the emotion task, was an outlier with the least reproducibility given the scan duration, possibly indicating that scan durations at or below 2 min are inadequate to construct meaningful functional connectivity measurements.

### Individual differences between subjects compared for task and resting state acquisitions

The results above show that systematic differences in functional connectivity should be effected when analyzing connectivity measurements obtained during different task paradigms. But if data for two subjects are acquired with the same task, can it be expected that intersubject differences in connectivity will be similar regardless of what task is performed?

**Figure 5:**
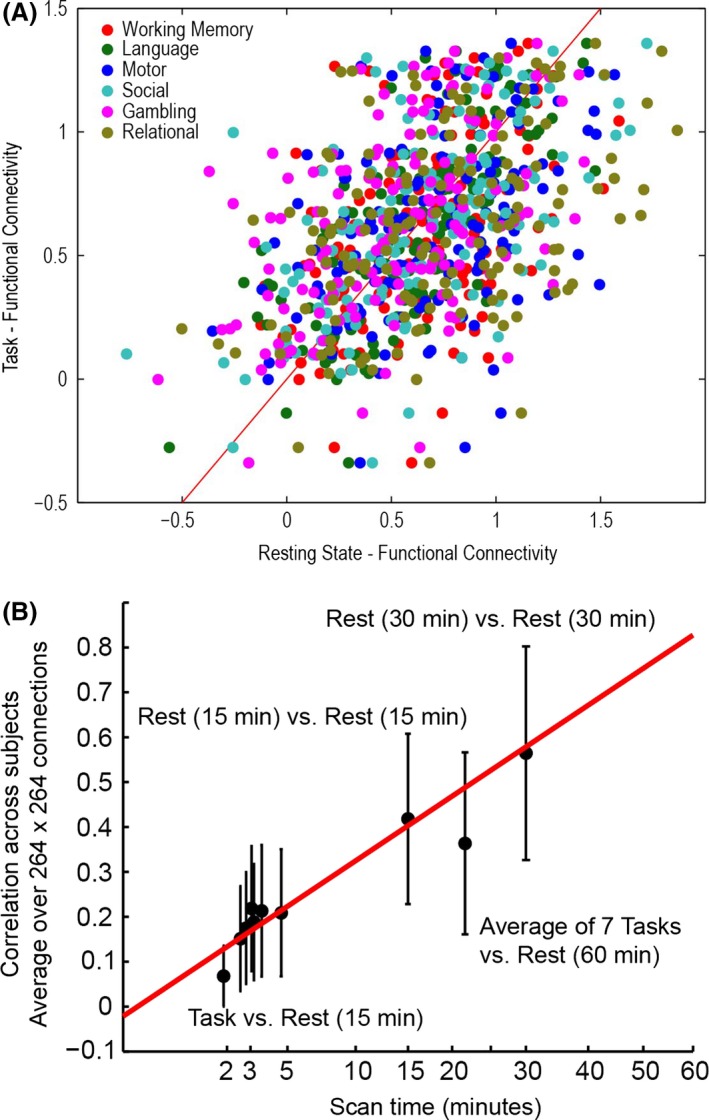
Preservation of individual differences in connectivity at task and rest. (A) One representative connection (left ACC to left anterior insula) showing for 476 subjects comparison of functional connectivity acquired during 6 tasks (1 acquisition) versus rest (60 min). (B) For each connection, the correlation across 476 subjects was computed for 264 × 264 ROIs. Error bars show standard deviation across connections. Correlation across subjects improves with the square root of imaging time of the shortest sequence.

To address this question, we measured the correlation coefficient across subjects for each individual connection acquired during two different task or resting acquisitions. This probes whether subjects that show particularly strong connectivity in a given connection might also show relatively strong connectivity for the same connection during a different task compared to other subjects. As an example, Figure 5A shows a single connection from the left anterior cingulate cortex to the left anterior insula correlation across all subjects, acquired during each task compared to acquisition during resting state. The significant correlation across subjects for a specific connection between rest and task is evidence that individual differences in connectivity persist during rest and task acquisitions. Regardless of which task was performed, differences in connectivity for that individual compared to other subjects may be expected to be similar regardless of the task condition. In other words, the same connections tend to show higher or lower functional connectivity in the same subjects whether data were acquired during a resting state or task condition.

This finding was observed not just for the single connection shown in Figure 5A, but generally for all connections studied. To obtain more global estimates of whether individual differences in connectivity persist across task acquisition conditions, we calculated the Fisher‐transformed correlation across subjects.

For *n* = 264 ROIs and *m* = 476 subjects, let *rsfc(ROI i, ROI j, task k, subject m)* represent the Fisher‐transformed resting state functional connectivity for a given subject *m* between ROI *i* and ROI *j* and task acquisition condition *k*. This task acquisition condition could represent functional connectivity calculated from data acquired during either a task or resting state acquisition. Then the mean functional connectivity for connection *ij* and task acquisition condition *k* across subjects may be represented as $\bar{rsfc(ROI\;t,ROI\;j,task\;k)}$ and the standard deviation for functional connectivity across subjects can be represented by *S*_*ij*,*k*_ Then *let r*_*ij*_ represent the Pearson correlation coefficient between ROI *i* and ROI *j* across subjects for task acquisition conditions *k*_1_ and *k*_2_:

$$\begin{array}{cc} r_{ij,k_{1},k_{2}} & =\frac{1}{475}\sum_{m=1}^{476}\left(\frac{rsfc\left(i,j,k_{1},m\right)-\bar{rsfc(i,j,k_{1})}}{S_{i,j,k_{1}}}\right)\\ & \left(\frac{rsfc\left(i,j,k_{2},m\right)-\bar{rsfc(i,j,k_{2})}}{S_{ij,k_{2}}}\right) \end{array}$$

This correlation coefficient was Fisher‐transformed and averaged across all connections ij to give the mean correlation between any two task acquisition conditions (comparing either 2 runs of the same task or 2 runs of different tasks) shown in Figure 5B.

The correlation across subjects improved with the square root of imaging time of the shortest sequence. Figure 5B shows that the correlation across subjects averaged over many such connections improves with the length of scan duration of the task. It may be that variations in individual differences may be the result of different scan lengths. The relevant factor appears to be not which task was performed, but of what duration the scan was.

### Effects of scan duration versus number of volumes on reliability

**Figure 6:**
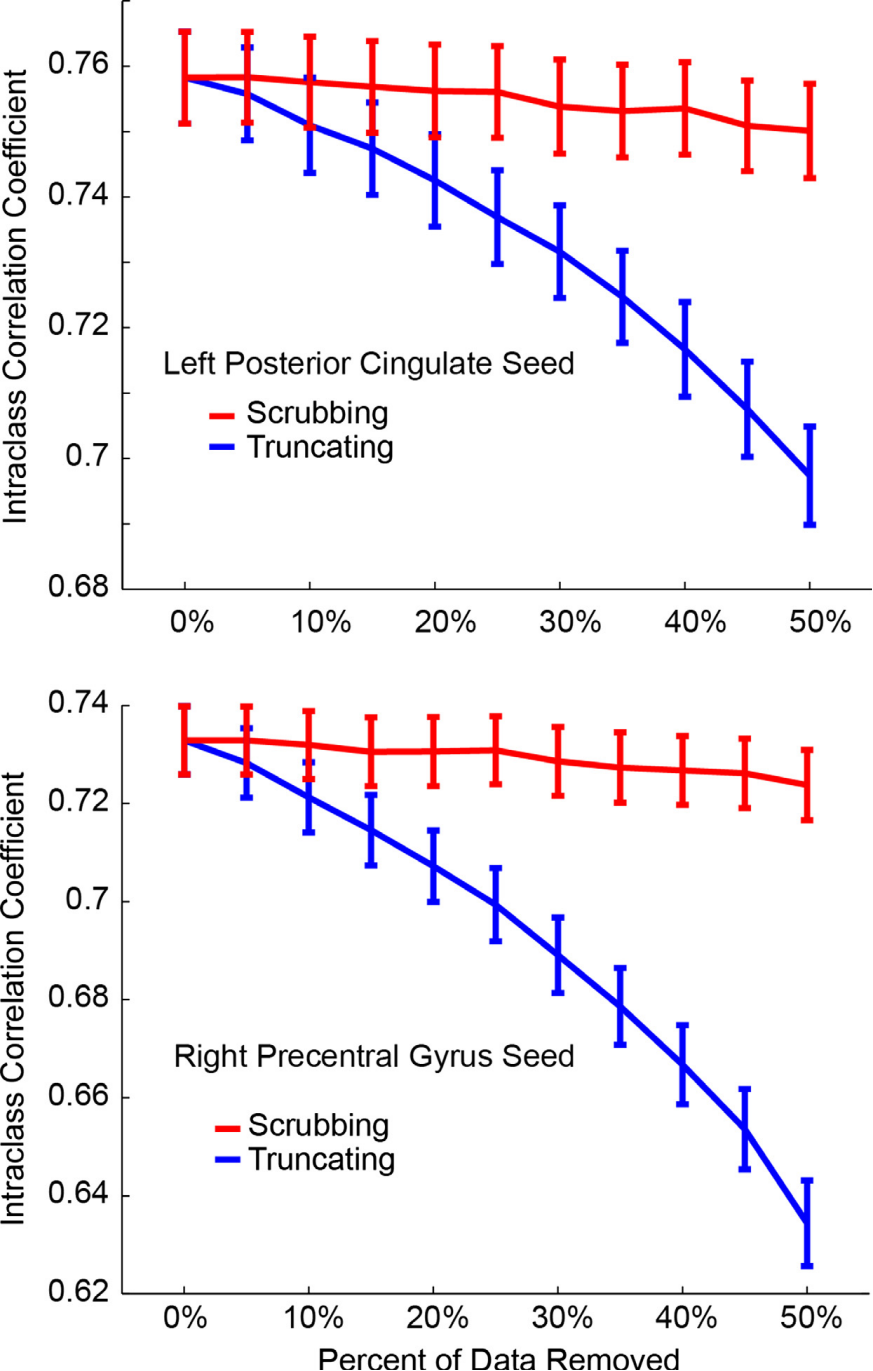
Effect of two strategies of data removal on reliability is shown. Intraclass correlation coefficient is shown for functional connectivity measurements from two seeds (left posterior cingulate, above; right precentral gyrus, below) to 264 ROIs after sequentially removing up to half of the volumes in the time series. Red curves (scrubbing) represent removal of randomly selected points in the time series. Blue curves (truncating) represent inclusion of timepoints from the beginning of the scan. Reliability is only minimally affected for scrubbing, even with removal of half of the data points. However, truncating the time series with removal of time points from the end of the time series shows much larger effects on reliability of functional connectivity measurements.

Intraclass correlation coefficient was calculated for each ROI in each subject with “simulated scrubbing” where randomly selected data volumes were removed showed almost no effect on reliability. However, truncation of the time series by a similar number of volumes had dramatic effect on reliability, shown in Figure 6, indicating that the key factor affecting reliability is scan duration, not necessarily the number of volumes used for analysis.

## Discussion

We report an analysis of data from the Human Connectome Project 500 Subject Release that reliability and reproducibility of multiband functional connectivity is determined by scan duration of acquisition. More specifically, reliability and reproducibility can be estimated by 1 over the square root of imaging time. This relationship was true for data obtained acquired during resting state as well as for data acquired during each of seven task paradigms. Functional connectivity results acquired during tasks were significantly modulated by the timing of the task paradigm, likely representing artificial synchrony of brain regions coactivated by the task. Nevertheless, individual differences in connectivity between subjects were similar regardless of the particular task performed, and these differences also become more reliable as a function of imaging time. Scan duration, not necessarily more volumes due to faster imaging sequences, was the critical factor in achieving higher reliability.

### Effects of data processing on reliability and reproducibility

One direction to improve reliability and reproducibility of functional connectivity measures is standardization of data processing. The HCP MRI data (Van Essen et al. 2013) are preprocessed with FIX ICA analysis (Griffanti et al. 2014), which detects consistent spatial components and separates signal from noise with functional networks being identified through their shared time courses. To analyze only signal from gray matter, we additionally performed regression of the signal derived from white matter and/or CSF voxels (Weissenbacher et al. 2009). We show that functional connectivity maps from the FIX ICA method have similar spatial distribution but systematically reduced functional connectivity in resting state and in task acquisitions as compared to white matter, CSF and motion correction preprocessing strategies (See Appendix S1).

Improvement in reliability and reproducibility may be achieved by addressing variability factors based upon the preprocessed datasets acquired with advanced resting fMRI sequences, such as the HCP datasets utilized in this study. Large‐sample multimodal test–retest neuroimaging datasets would be valuable, as the available small sample studies would be statistically powered with a large test–retest sample. To help achieve these objectives, the Consortium for Reliability and Reproducibility has released a multisite database to address the variability in data acquisition, experimental designs, and analytic methods (Zuo et al. 2014). The CoRR provides a data platform, International Neuroimaging Data‐sharing Initiative (Milham 2012), by which researchers can explore the reliability and reproducibility of fMRI indices and connectomics‐based measures, which, then, may be generalized and used as biomarkers.

### Factors affecting reliability and reproducibility of functional connectivity

For meaningful clinical application, the impetus of fMRI research is to characterize the many sources of variations in the functional connectome across disorders and individuals and their impact on resting state fMRI measures. Low intraindividual variability is required for high test–retest reliability (Zuo and Xing 2014). Reliable single subject metrics and test–retest reliability are critical for the development of imaging biomarkers for the detection and early intervention in CNS disorders as well for evaluating developmental and senescent changes. Interindividual differences may be related to intrinsic functional architecture, whereas intraindividual variability may in part be due to non‐neural factors such as scan conditions (Yan et al. 2009), head motion (Power et al. 2012; Van Dijk et al. 2012; Yan et al. 2013a), physiologic noise (Birn et al. 2008; Chang and Glover 2009a,b; Chang et al. 2009), and data analysis/standardization strategies (Yan et al. 2013b). Gorgolewski et al. found that motion correlated with stimuli had a non‐negligible influence on single‐subject reliability more than absolute motion (Gorgolewski et al. 2013). Preprocessing strategies may also impact reliability and reproducibility. Global signal regression, for instance, has been shown to lower the test–retest reliability of local functional homogeneity (Zuo et al. 2013). Although functional connectomes have features that are temporally stable or exhibit statistically negligible intraindividual variability, neural and non‐neural factors likely contribute to the dynamic changes in resting‐state functional connectivity (Hutchison et al. 2013).

Considering the spatial distribution of reliability and reproducibility may also be important in planning clinical functional connectivity tests. Our data indicate that reliability decreases significantly when considering connections involving deep gray nuclei, regions near large sulci, and brain regions at the gray‐white junction as opposed to cortical regions. A limitation of the fine, granular parcellation of connectivity analysis in this study may be affected by many factors that are difficult to control, such as voxel averaging particulary at tissue boundaries and functional‐structural discordance. Future studies could explore the reliability and reproducibility of these results with data‐driven approaches to definition of larger ROIs. If clinical assays are anticipated using these regions, it may require longer scans or more robust clinical differences to produce metrics that can distinguish diagnosis, prognosis, or treatment effects.

### Prior results on task‐evoked functional connectivity

It has been conventional to acquire functional connectivity data during a resting or “no‐task” state with the presumption that comparing data across subjects may be possible without concerns about task performance. Yet the resting state is not necessarily a single state with unconstrained brain activity, but rather another task, albeit one with many parameters unknown to the experimenters. Across reported studies “resting state” scans are variably performed with eyes open, eyes closed, with visual fixation points or without, and with as many different sets of subject instructions as there are investigators. Each of these factors can substantially affect results (Zou et al. 2009; Patriat et al. 2013). Lying awake in a scanner is a cognitive exercise and can be anxiety‐provoking. The resting scan may be performed differently for different patient groups with interactions between diagnosis and how the “resting” task is performed. Should one focus on breathing, on recollecting events, on meditation? Drowsiness or sleep can dramatically affect functional connectivity patterns (Larson‐Prior et al. 2011; Tagliazucchi and Laufs 2014; Yeo et al. 2015). Evidence is mounting from simultaneous fMRI/EEG reports that many or most subjects enter early stages of sleep within minutes of the onset of a resting state scan, and generally have poor insight as to their state of arousal (Tagliazucchi and Laufs 2014). All these problems are compounded if it becomes necessary to collect long scans on the order of an hour or more rather than a few minutes as is the current practice.

Studies have established that functional networks and consistent patterns of activation elicited via task performance are recapitulated by resting‐state fMRI (Biswal et al. 1995; Smith et al. 2009; Biswal et al. 2010; Sporns 2014; Yeo et al. 2011; Zuo et al. 2012; Buckner et al. 2013; Smith et al. 2013). Mennes et al. (2011) and Zou et al. (2013) have demonstrated systematic differences in group‐mean functional connectivity acquired during task and rest and during different tasks, with higher connectivity in task‐relevant brain regions. Yet there is also literature that task modulation of functional connectivity may be more complex than enhancement of connectivity in engaged brain regions. While much of the variance of a task‐based functional MRI acquisition can be attributed to “cognitive noise” associated with spontaneous brain activity (Gorgolewski et al. 2013), group differences in connectivity seen during resting state acquisition may be similar to those seen during task acquisition.

When the connectivity for five major functional networks (salience, default mode, visual, sensorimotor, dorsal attention) was evaluated, a similar spatial distribution between task and rest acquisitions was seen. Although the group mean functional connectivity showed modulation with the task, connectivity differences appeared to be preserved across individuals. Furthermore, the different tasks, including the timing and structure, changed the pattern of connectivity in a systematic way. The higher correlation for a specific connection between rest and task across subjects demonstrated in our study is further evidence that individual differences in connectivity persist during rest and task acquisitions. Individual differences in connectivity may be expected to be similar regardless of the task condition for which the data were acquired. The differences may be due to intrinsic differences in individual's brain activity, whether that is due to structural white matter connections and/or greater gray matter volume.

### Effects of scan duration on reliability and reproducibility of functional connectivity

The results from this study highlight that longer scan times are needed to acquire data on single subject networks and information on the connectivity from single brain regions. Specifically, the reliability and reproducibility of functional connectivity a single connection as well as across subjects for resting and task acquisition were demonstrated to be a linear function of the square root of imaging time (Van Dijk et al. 2010; Anderson et al. 2011). Van Dijk et al. and Shehzad et al. demonstrated that moderately reliable estimates could be obtained from a single 5‐min run (Shehzad et al. 2009; Van Dijk et al. 2010), which was shown to achieve 50% reliability by Zuo et al. (2013). This large measurement variability with shorter imaging times will likely limit the accurate classifications of pathologies.

Although “ensembles” of connections can be used, they may not allow for the distinction between subtypes or limit the development of connectivity endophenotypes of clinical syndromes. Previous results have shown that within a single subject, the ability of an automated machine‐learning classifier to discriminate differences in connectivity attributable to a task increases with longer imaging time (Anderson et al. 2011). In their study looking at correlation measurements between any two small regions of interest within a subject and at any connection in a healthy population as a function of time, Anderson et al. found that individual and population connectivity can be reliably discerned at 15 min imaging time with increased reliability at <=4 h (Anderson et al. 2011). Imaging time >25 min is necessary to identify a single connection in an individual as different from the group reliably (Anderson et al. 2011).

Zuo et al. demonstrated that a longer scan time can improve the test–retest reliability of the functional homogeneity metric (Zuo et al. 2013). In contradistinction to Van Dijk et al. who found that reliability measures decreased with the square root of imaging time (Van Dijk et al. 2010), our study not only demonstrated increased reliability and reproducibility of a single connection for a single subject for both task and resting acquisitions with increased imaging time but also increased correlation across subjects with the square root of imaging time of the shortest sequence. A recent study by Birn et al. showed that test–retest reliability and across‐session similarity of resting‐state functional connectivity are greatly improved by increasing the scan lengths from 5 min up to 13 min, particularly if the scans are acquired during the same session (Birn et al. 2013). They suggest that the improved reliability with the increase in the number of volumes as well as the increase in the length of time over which these volumes was acquired may be because resting‐state functional connectivity estimates are modulated by slow frequency dynamics, with cycles on the order of several minutes (Birn et al. 2013).

Our results that incorporate random removal of brain volumes (scrubbing) versus truncation of scans corroborate that the primary driver of reliability is the length of scan. Random scrubbing up to 50% of the datapoints had very little effect on reliability while truncating the data to a similar degree had a much larger effect. Since functional connectivity measures relatively slow (<0.08 Hz) changes, removal of random time points did not appear to apreciably effect the results, but shorter scan durations allowing less time for the brain to sample different states had a much larger effect on reliability. Resting state connectivity metrics are ultimately a measure of synchrony and are very sensitive to specific mental processes that are taking place during the scan. Changes in mood, content of thought, and arousal state are relatively slow processes, and sampling a short duration of brain connectivity is likely to be skewed toward a given state. This improves with data collated from multiple scan sessions or longer imaging times.

Longer imaging times for measuring functional connectivity may provide increased reliability and reproducibility, but the limiting factor is the subject's ability to tolerate prolonged scanning. There are the realistic issues of maintaining wakefulness and stillness, discomfort in the prolonged prone position and pathology‐related hindrances. Acquiring functional connectivity data during stimulation or task paradigms will enable longer scan durations with better characterization of brain states during scanning. Task acquisition may facilitate holding a subject's attention with less risk of falling asleep during the scan and, as shown by our results, there are preserved individual differences in functional connectivity at task and rest acquisitions.

## Conclusions

Clinical application of functional connectivity research to individual patients and to the development of disease classifications requires test–retest reliability and reproducibility. Our study found systematic differences in group‐mean connectivity acquired during task and rest and preserved individual differences in connectivity during rest and task acquitisions due to intrinsic differences in an individual's brain activity. Reproducibility in task and rest acquisitions is similar, although the group mean functional connectivity will show modulation with the task. We also showed that longer scan times are needed to acquire data on single subject networks and information on the connectivity between focused brain regions. Our results illustrated that the primary driver of reliability and reproducibility is the length of the scan, with longer scan times allowing the brain to sample a broader range of cognitive states with improved reliability and reproducibility.

## Conflict of Interest

None declared.

## Supporting information


**Figure S1.** Effect of preprocessing strategy on group mean connectivity for 6923 × 6923 ROI pairs.

**Figure S2.** Systematic changes in connectivity associated with FIX ICA consist of reduced connectivity but similar spatial distribution across five different seeds.

**Appendix S1.** Effect of preprocessing methods on fMRI data analysis.
