# Supplementary figures and images for: Reliability and reproducibility of individual differences in functional connectivity acquired during task and resting state
Source: Brain Behav. 2016 Mar 30;6(5):e00456. doi: 10.1002/brb3.456 (PMC4814225; doi:10.1002/brb3.456)

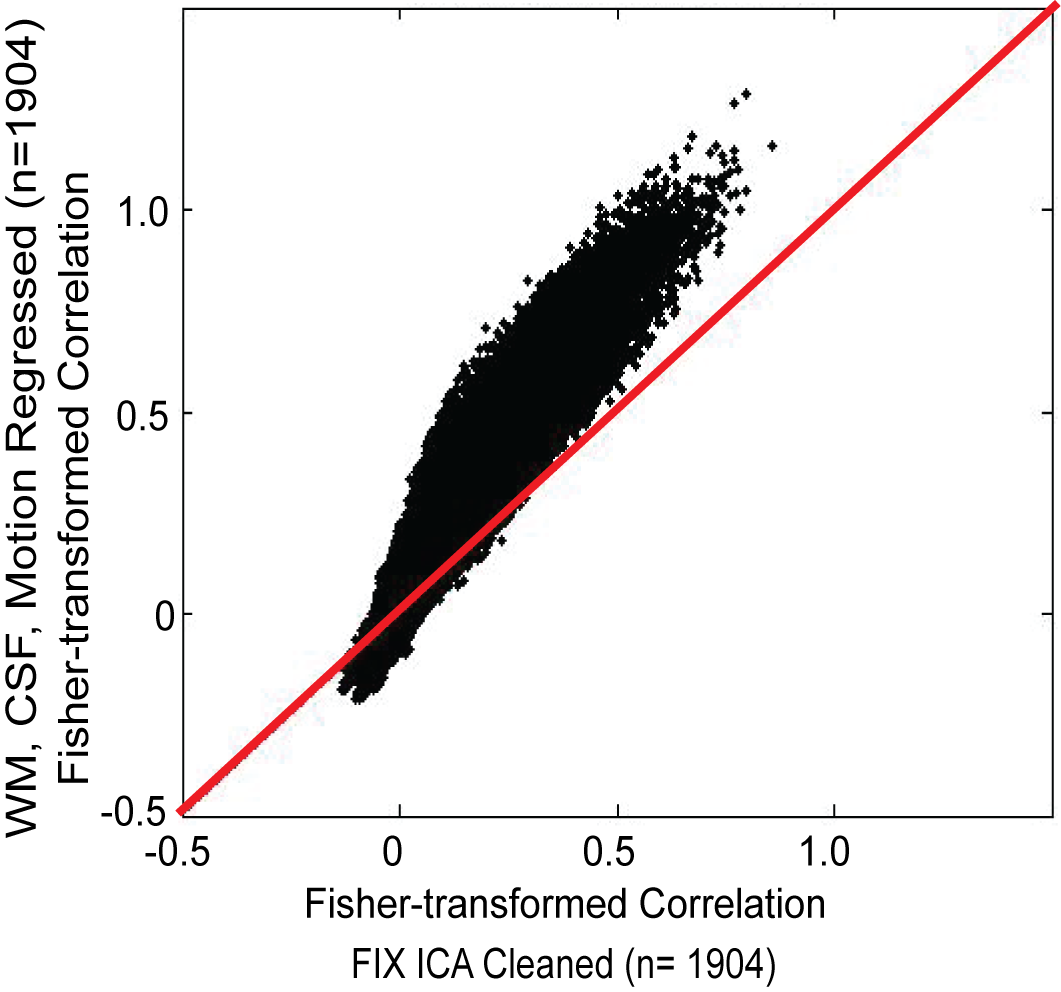

Supplement: Supplementary file 1 — Figure S1. Effect of preprocessing strategy on group mean connectivity for 6923 × 6923 ROI pairs. [file BRB3-6-e00456-s001.tiff]

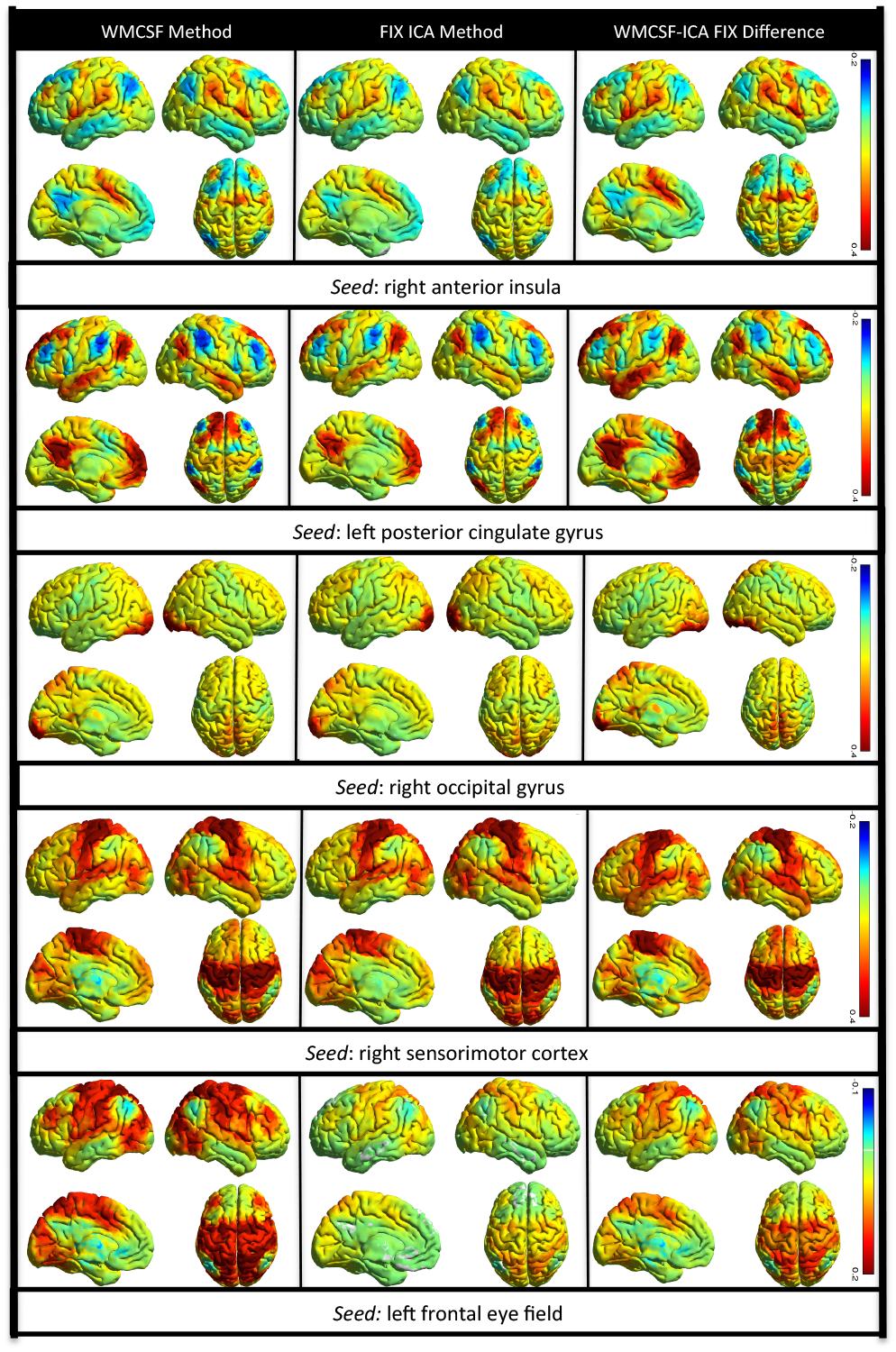

Supplement: Supplementary file 2 — Figure S2. Systematic changes in connectivity associated with FIX ICA consist of reduced connectivity but similar spatial distribution across five different seeds. [file BRB3-6-e00456-s002.tif]
