# Supplementary material for: Reliability and reproducibility of individual differences in functional connectivity acquired during task and resting state
Source: Brain Behav. 2016 Mar 30;6(5):e00456. doi: 10.1002/brb3.456 (PMC4814225; doi:10.1002/brb3.456)
Supplement: Supplementary file 3 — Appendix S1. Effect of preprocessing methods on fMRI data analysis. [file BRB3-6-e00456-s003.docx]

**Supplementary Material**:

**Effect of Preprocessing methods on fMRI data analysis.**

As part of the HCP 500 Subjects Release, each of the 476 subjects has undergone 4 MRI sessions with these anatomic and functional sequences: diffusion imaging, resting-state fMRI, task-evoked fMRI, and T1- and T2-weighted MRI for structural and myelin mapping. BOLD fMRI images were performed using multiband technique with TR: 720 ms, TE: 33.1 ms, 72 slices, 2x2x2 mm spatial resolution, 1200 volumes for resting fMRI sequences. Detailed description of the exact MRI specifications can be found at <http://www.humanconnectome.org/documentation/S500/HCP_S500+MEG2_Release_Appendix_I.pdf>**.** The HCP minimal preprocessing structural pipeline, FreeSurfer, produces an undistorted “native” structural volume space for each subject, aligns the T1-weighted and T2-weighted images, performs a B1 (bias field) correction, and registers the subject’s native structural volume space to MNI space (Glasser et al., 2013). EPI distortion correction with gradient field maps is also performed. With the functional pipeline, fMRIVolume, spatial distortions are removed, volumes are realigned to compensate for subject motion, fMRI data is registered to the structural images, the bias field is reduced, the 4D image is normalized to a global mean, and the data is masked with the final brain image.

Improved resting fMRI results with 2-3 mm isotropic resolution are achieved with the multiband technique in which many slices are acquired in the time of a single echoplanar imaging echo train and a single contrast preparation period. Additionally, TR has been reduced to obtain more signal to noise ratio (SNR) and improved temporal resolution for task-related fMRI. The improved temporal resolution is intended to help delineate differences in the time course of task activation/deactivation for the particular brain region and/or task. Other efforts to optimize acquisition parameters in study are aimed to maximize the number of functional parcels that can be reproducibly distinguished from one another and the network connections between these parcels (Van Essen et al., 2012). The information from the task-related fMRI sequences is used to help identify functionally distinct brain parcels that can validate and interpret connectivity results from the resting fMRI and diffusion MRI. The network connectivity in a task context can be compared to resting fMRI connectivity results. Together, the task-related fMRI and resting fMRI may be used to predict individual differences in behavior and genetic influences. Phase I of the HCP includes predominantly block design paradigms with tasks that measure visual-motor processes (such as retinotopy, motor strip mapping, both biological and non-biological motion), cognitive processes (such as working and episodic memory, attention, language, stimulus category representations) and affective/social processes (reward and punishment based decision making, emotion recognition, and social cognition) (Van Essen et al., 2012). The tasks assessing multiple networks simultaneously were used in the HCP protocols. Tasks proven as localizers in individual subjects or with evidence for reliability across subjects or within subjects across time were chosen.

A portion of this study involved the analysis of multiband BOLD data from the 476 subjects using both FIX ICA cleaned and minimally preprocessed data with WM, CSF and motion parameter regression. Preprocessing of fMRI data can affect the data analysis. The FIX ICA method uses independent component analysis (ICA) with automated noise component selection and regression of noise components from the data (Griffanti et al., 2014). Because noise component selection is derived from data acquired in a resting state sample, it is uncertain whether this method may result in asymmetric performance for resting state versus task acquisition. In light of this, a uniform processing strategy was implemented in this study on the minimally preprocessed HCP data for both the task and resting state acquisition.

**Methods:**

Minimally preprocessed data (Glasser et al., 2013) were treated with the following preprocessing steps:

1. A gray matter mask was compiled from skull stripped BOLD images for all 476 subjects showing voxels where an a priori gray matter mask (grey.nii, SPM 12b) were inside the brain for 95% of subjects. This image was parcellated into 6923 non-overlapping 5-mm diameter ROIs covering the cortical and subcortical gray matter. The 6923 ROIs were chosen to represent a parcellation of the gray matter with spatial resolution of 5 mm. Specifically, each voxel was tested in sequence beginning with the inferior left voxel in the cerebellum. If a voxel was greater than 5 mm distant to voxels already selected, then this voxel was included in the set of ROI center coordinates. When all voxels had been tested, 6923 voxels remained, and gray matter voxels were parcellated based on which of the 6923 center coordinates was closest to a given voxel.
2. The MPRAGE image for each subject was segmented using SPM12b into gray matter, white matter, and CSF images, and a mask was created for each subject by thresholding these images at 0.5. This mask was degraded for CSF and WM by eliminating all voxels that were not surrounded on all sides by CSF and WM voxels in the mask.
3. A bandpass filter was applied (idealfilter.m, MATLAB) between 0.001 and 0.1 Hz, and each time series was subjected to a linear detrend operation in conjunction with the WM, CSF, and motion regression (each time series and covariate was detrended and bandpass filtered prior to regression).

FIX ICA cleaned BOLD resting state data (Griffanti et al., 2014) were used without additional preprocessing steps, to calculate Fisher transformed correlation coefficients for the same 6923 x 6923 ROIs for each resting state acquisition.

The Fisher transformed correlation coefficients were obtained for each pair of 6923 x 6923 ROIs, which represent the functional connectivity and which were analyzed for the spatial resolution of the functional networks. To compare canonical network patterns, 5 of the 6923 ROIs were selected a priori to represent distinct functional networks: Seed 1, right anterior insula (salience network, MNI coordinates: x=42, y=12, z=10); Seed 2, left posterior cingulate (default mode network, MNI coordinates: x=-4, y=-50, z=30); Seed 3, right occipital pole (visual network, MNI coordinates: x=14, y=-98, z=-10); Seed 4, right precentral gyrus (sensorimotor network, MNI coordinates: x=38, y=-22, z=60); Seed 5, left frontal eye field (dorsal attention network, MNI coordinates: x=25, y=-4, z=50). Connectivity of the other 6922 ROIs to each of the 5 seed ROIs was used to obtain snapshots of core canonical resting state networks with different preprocessing strategies.

**Results:**

In this study, the WM,CSF, motion-regressed method resulted in slightly higher Fisher-transformed correlations of group mean connectivity for each pair of 6923 x 6923 ROIs for each of the 4 resting state sequences as compared to the correlation coefficients for the same 6923 x 6923 ROIs for each resting state acquisition FIX ICA-cleaned BOLD resting state data. The overall spatial pattern of seed-based functional connectivity between the diffferent preprocessing strategies was similar. (Supplementary Section Figure 1)

The effect of the FIX ICA method preprocessing strategy on canonial network patterns in resting state and in task acquisition was systematically reduced connectivity but preserved spatial distribution across 5 distinct functional networks: salience, default mode, visual, sensorimotor, and dorsal attention (Supplementary Section Figure 2).

**Discussion:**

Functional magnetic resonance imaging (fMRI) uses blood oxygen level dependent (BOLD) signal to indirectly measure neuronal activity; however, this BOLD signal includes that from white matter, cerebrospinal fluid (CSF) pulsation and heart, respiratory and head motion. FMRI data is extremely sensitive to movement, even when it is less than 1 mm. Additionally, low frequency drifts (Smith et al., 1999) and high amplitude oscillations due to pulse effects (Jiang et al., 2002) also contribute to the BOLD signal. This is particularly important in resting-state fMRI, in which the artifacts can overlap spatially or spectrally with resting state networks. In resting-state fMRI, the temporal similarity between BOLD signals in different regions of the brain at rest is assessed to reveal synchronous neural activity. The statistical dependence among the time series across time without any consideration for causality characterizes functional connectivity (Friston, 2011). The use of multiband, fast-TR, pulse sequences has been proposed as a strategy to mitigate effects of head motion and physiological noise by “freezing” head motion and eliminating physiological artifacts such as heart rate being aliased into low frequency ranges of interest to functional connectivity (Feinberg et al., 2010; Glasser et al., 2013; Griffanti et al., 2014; Moeller et al., 2010; Setsompop et al., 2012; Van Essen et al., 2013; Xu et al., 2012).

One direction to improve reliability of functional connectivity measures is standardization of data processing. Regressing out the signal derived from white matter and/or CSF voxels is a proposed method to analyze only signal from grey matter (Weissenbacher et al., 2009), which was implemented in this study. As such, an important part of this study was to determine the effect of preprocessing strategies on BOLD signal. The HCP MRI data (Van Essen et al., 2013) is preprocessed with FIX ICA analysis (Griffanti et al., 2014). Independent component analysis is a data-driven approach to recover source signals from a mixture with unknown mixing coefficients and is based on the assumed statistical independence of the source signals (McKeown et al., 2003). The ICA method detects consistent spatial components and separates signal from noise. Functional networks can be identified through their shared time courses. ICA components reflect the data and, therefore, they are sensitive to any movement including abrupt changes and slow, linear drifts (McKeown et al., 1998). As some components reflect purely BOLD signal and others artifacts, the latter may be regressed out of the data. Manual identification of these artifactual components is tedious, prone to operator error and requires expertise in the spatial and temporal characteristics of signal and noise fluctuations. Among the several automated ICA approaches for cleaning fMRI data, the FMRIB ICA-based X-noiseifier analysis (FIX ICA) method achieves the balance between noise removal and signal loss by regressing out the full space of motion-related fluctuations and only the unique variance of the artifactual ICA components (Griffanti et al., 2014).  FIX ICA analysis may accentuate inter-subject variability due to its sensitivity to the underlying data structure.

To study the effect of preprocessing strategy, the minimally processed HCP MRI data (Glasser et al., 2013; Van Essen et al., 2013) was also analyzed with white matter, CSF and motion correction method. This data was co-registered with anatomic MPRAGE sequences, and all motion-corrected images were spatially normalized to the Montreal Neurologic Institute echo-planar imaging template. We show that functional connectivity maps from the FIX ICA and white matter, CSF and motion correction preprocessing strategies have similar spatial distribution although there is systematically reduced functional connectivity in resting state and in task acquisitions.

Conclusion:

The analysis of multiband BOLD data from the HCP 500 Subjects Release with different preprocessing methods, FIX ICA versus WM, CSF and motion regression method, showed differences in the amplitude of correlations of group mean connectivity but not in the spatial distribution.

**Figure Legends:**

Figure 1: Effect of preprocessing strategy on group mean connectivity for 6923 x 6923 ROI pairs. Although the WM,CSF, motion-regressed method resulted in slightly higher Fisher-transformed correlations of group mean connectivity as compared to the correlation coefficients for the same ROIs for each resting state acquisition FIX ICA-cleaned BOLD resting state data, the overall pattern between the diffferent preprocessing strategies was similar.

Figure 2: Systematic changes in connectivity associated with FIX ICA consist of reduced connectivity but similar spatial distribution across 5 different seeds. The left column shows seed-based functional connectivity for data processed using WM, CSF, and motion regression technique. The middle column reflects seed-based functional connectivity for data processed using FIX ICA method. The right column shows the difference between data processed using WMCSF and FIX ICA methods. Color scale bars show mean Fisher-transformed correlation.

References:

Feinberg, D.A., Moeller, S., Smith, S.M., Auerbach, E., Ramanna, S., Gunther, M., Glasser, M.F., Miller, K.L., Ugurbil, K., Yacoub, E., 2010. Multiplexed echo planar imaging for sub-second whole brain FMRI and fast diffusion imaging. PLoS One 5, e15710.

Friston, K.J., 2011. Functional and effective connectivity: a review. Brain Connect 1, 13-36.

Glasser, M.F., Sotiropoulos, S.N., Wilson, J.A., Coalson, T.S., Fischl, B., Andersson, J.L., Xu, J., Jbabdi, S., Webster, M., Polimeni, J.R., Van Essen, D.C., Jenkinson, M., Consortium, W.U.-M.H., 2013. The minimal preprocessing pipelines for the Human Connectome Project. Neuroimage 80, 105-124.

Griffanti, L., Salimi-Khorshidi, G., Beckmann, C.F., Auerbach, E.J., Douaud, G., Sexton, C.E., Zsoldos, E., Ebmeier, K.P., Filippini, N., Mackay, C.E., Moeller, S., Xu, J., Yacoub, E., Baselli, G., Ugurbil, K., Miller, K.L., Smith, S.M., 2014. ICA-based artefact removal and accelerated fMRI acquisition for improved resting state network imaging. Neuroimage 95, 232-247.

Jiang, H., Golay, X., van Zijl, P.C., Mori, S., 2002. Origin and minimization of residual motion-related artifacts in navigator-corrected segmented diffusion-weighted EPI of the human brain. Magn Reson Med 47, 818-822.

McKeown, M.J., Hansen, L.K., Sejnowsk, T.J., 2003. Independent component analysis of functional MRI: what is signal and what is noise? Curr Opin Neurobiol 13, 620-629.

McKeown, M.J., Jung, T.P., Makeig, S., Brown, G., Kindermann, S.S., Lee, T.W., Sejnowski, T.J., 1998. Spatially independent activity patterns in functional MRI data during the stroop color-naming task. Proc Natl Acad Sci U S A 95, 803-810.

Moeller, S., Yacoub, E., Olman, C.A., Auerbach, E., Strupp, J., Harel, N., Ugurbil, K., 2010. Multiband multislice GE-EPI at 7 tesla, with 16-fold acceleration using partial parallel imaging with application to high spatial and temporal whole-brain fMRI. Magn Reson Med 63, 1144-1153.

Setsompop, K., Gagoski, B.A., Polimeni, J.R., Witzel, T., Wedeen, V.J., Wald, L.L., 2012. Blipped-controlled aliasing in parallel imaging for simultaneous multislice echo planar imaging with reduced g-factor penalty. Magn Reson Med 67, 1210-1224.

Smith, A.M., Lewis, B.K., Ruttimann, U.E., Ye, F.Q., Sinnwell, T.M., Yang, Y., Duyn, J.H., Frank, J.A., 1999. Investigation of low frequency drift in fMRI signal. Neuroimage 9, 526-533.

Van Essen, D.C., Smith, S.M., Barch, D.M., Behrens, T.E., Yacoub, E., Ugurbil, K., Consortium, W.U.-M.H., 2013. The WU-Minn Human Connectome Project: an overview. Neuroimage 80, 62-79.

Van Essen, D.C., Ugurbil, K., Auerbach, E., Barch, D., Behrens, T.E., Bucholz, R., Chang, A., Chen, L., Corbetta, M., Curtiss, S.W., Della Penna, S., Feinberg, D., Glasser, M.F., Harel, N., Heath, A.C., Larson-Prior, L., Marcus, D., Michalareas, G., Moeller, S., Oostenveld, R., Petersen, S.E., Prior, F., Schlaggar, B.L., Smith, S.M., Snyder, A.Z., Xu, J., Yacoub, E., Consortium, W.U.-M.H., 2012. The Human Connectome Project: a data acquisition perspective. Neuroimage 62, 2222-2231.

Weissenbacher, A., Kasess, C., Gerstl, F., Lanzenberger, R., Moser, E., Windischberger, C., 2009. Correlations and anticorrelations in resting-state functional connectivity MRI: a quantitative comparison of preprocessing strategies. Neuroimage 47, 1408-1416.

Xu, J., Moeller, S., Strupp, J., Auerbach, E., Feinberg, D., Ugurbil, K., Yacoub, E., 2012. Highly Accelerated Whole Brain Imaging Using Aligned-Blipped-Controlled-aliasing Multiband EPI. Proc. Int. Soc. Mag. Reson. Med 20:2306.
